# Spatial–Temporal Dynamics and Co-Occurrence Networks of Zooplankton Community Structure in a Large Shallow Reservoir Under the Background of Ecological Restoration

**DOI:** 10.3390/ani16132065

**Published:** 2026-07-04

**Authors:** Ting Yuan, Jiangqianhui Qi, Mantang Xiong, Geng Huang, Kui Zhang, Lianfeng Zhou, Feng Chen

**Affiliations:** Key Laboratory of Ecological Impacts of Hydraulic-Projects and Restoration of Aquatic Ecosystem of Ministry of Water Resources, Institute of Hydroecology, Ministry of Water Resources & Chinese Academy of Sciences, Wuhan 430079, China; yuanting@mail.ihe.ac.cn (T.Y.); qjqh0515@163.com (J.Q.); xiongmantang@mail.ihe.ac.cn (M.X.); wangyoukl@126.com (G.H.); zhangkui@mail.ihe.ac.cn (K.Z.); zhoulf@mail.ihe.ac.cn (L.Z.)

**Keywords:** zooplankton, community structure, co-occurrence network, ecological restoration, Shilianghe Reservoir

## Abstract

Zooplankton are sensitive indicators of ecological change, but their community reorganization under early-phase restoration remains unclear. This study aims to reveal how their diversity, standing stock, and co-occurrence networks respond to early-phase restoration and identifies key environmental drivers, offering novel insights into restoration–plankton interplay. Shilianghe Reservoir, as the largest reservoir in Jiangsu Province, represents a typical case of restoration in the Huai River Basin that offers transferable insights for numerous eutrophic reservoirs. The study comprised eight surveys representing two annual cycles (July 2021–May 2022 and April 2023–January 2024). The 2023 sampling cycle showed lower zooplankton density and biomass but higher *α*-diversity than the 2021 sampling cycle. Co-occurrence network density and the size of the largest connected component decreased in spring and summer of the 2023 sampling cycle, suggesting community reorganization. Inflow tributaries supported the highest species richness and standing stock but showed looser interspecific associations. PERMANOVA analysis indicated that season explained the largest proportion of community variation, followed by sampling year and region. Environmental variables including DO, WT, Cond and TN significantly correlated with zooplankton community structure. Zooplankton community reorganization was characterized by reduced standing stock, increased diversity, and altered co-occurrence patterns, providing a mechanistic framework for using zooplankton as bioindicators in reservoir restoration assessments.

## Simple Summary

Zooplankton are sensitive indicators of environmental change and food-web regulation in freshwater ecosystems. The Shilianghe Reservoir, which is located in the Huai River Basin, is a typical case demonstrating initial restoration success. However, the response of the zooplankton community to water quality enhancement remains unclear. We investigated zooplankton composition, standing stock, diversity, co-occurrence associations and environmental drivers in this large shallow drinking-water reservoir in eastern China, based on two annual sampling cycles from 2021 to 2024. The results showed that temporal variation explained more community change than spatial zonation, while inflow tributaries supported high species richness and standing stock but relatively loose co-occurrence associations. Compared with the 2021–2022 cycle, the 2023–2024 cycle showed lower density and biomass but higher diversity, which suggested zooplankton community reorganization. These observed changes are temporally aligned with the implementation of restoration measures, though other environmental and stochastic factors may also contribute. Subsequent restoration efforts should focus on controlling external pollution inputs and regulating food-web structure. These findings provide a case-based framework for using zooplankton communities to evaluate ecological change in thousands of reservoirs undergoing ecological restoration in the Huai River Basin.

## 1. Introduction

Zooplankton are a key component of the food web in freshwater ecosystem, capable of altering phytoplankton community structure through top-down effects [1,2]. Meanwhile, zooplankton serve as natural prey for filter-feeding fish, most small fish and fish in early life stages, influencing fishery resources through bottom-up effects [3], acting as the energy link connecting primary producers such as phytoplankton with higher-trophic-level consumers such as fish [4,5]. In addition, due to their short life cycle, rapid turnover rate and high sensitivity to environmental changes, zooplankton are often used as an important indicator organism for assessing the trophic status and ecological health of water bodies [6,7]. Previous studies have demonstrated that environmental factors such as water temperature, nutrient concentrations, dissolved oxygen and water transparency can significantly affect the community composition, standing stock and diversity characteristics of zooplankton [6,8,9]. Particularly in large reservoirs, influenced by hydrodynamic processes, nutrient inputs and habitat characteristics, zooplankton communities typically exhibit pronounced spatiotemporal heterogeneity [10,11,12].

Co-occurrence network analysis, as a widely applied and highly valued research tool in ecological research, is based on random matrix theory [13]. By constructing modular networks of biological community data, it enables an in-depth analysis of the complex relationships within biological communities [14]. This method primarily relies on species occurrence probabilities at the community level and abundance data of individual species to construct an intuitive and visual network model [15,16]. The co-occurrence network model can accurately present both the functional characteristics of biological communities and their internal structural compositions within ecosystems. Through the network model, researchers are able to gain a deeper and more comprehensive understanding of the complex relationships among different species [17].

The inland freshwater bodies of China can be divided into seven major river basins, including the Yangtze River Basin and the Huai River Basin. Shilianghe Reservoir, which is located in the Huai River Basin, is the largest reservoir in Jiangsu Province. Its water function zoning is designated as the drinking water source area for Lianyungang City, which means its water quality is crucial to drinking water security. In the past few decades, the reservoir has been simultaneously affected by multiple factors including high-density cage aquaculture, overfishing and external pollution inputs, leading to prominent eutrophication in local water areas [18,19]. An ecological restoration project of Shilianghe Reservoir was initiated in 2019. However, the implementation of these measures progressed slowly, and it was not until 2021 when national-level ecological restoration efforts were comprehensively advanced that the ecological restoration measures in the Shilianghe Reservoir were fully carried out. With the implementation of a series of measures such as the regulation of illegal sand mining, aquaculture cage removal, a comprehensive fishing ban, sewage interception and treatment in surrounding villages and towns, restoration of estuarine wetlands, and filter-feeding fish (silver carp *Hypophthalmichthys molitrix* and bighead carp *Aristichthys nobilis*) stocking, the internal pollution load within the reservoir has been significantly reduced [20]. To date, a total of 35 million silver carp and bighead carp have been stocked in Shilianghe Reservoir [21].

There are approximately 8600 reservoirs in the Huai River Basin, a considerable number of which are undergoing ecological restoration measures. The Shilianghe Reservoir, as a typical case demonstrating early-phase restoration, can provide a reference for restoration measures and evaluation methods for other reservoirs undergoing ecological restoration in the Huai River Basin. As an important indicator organism for assessing the water quality status and ecosystem health of water bodies, zooplankton assemblages are inevitably altered by freshwater restoration operations. Current research on Shilianghe Reservoir mainly focuses on water quality, sediment and phytoplankton communities [18,19], while empirical studies spanning multiple years and providing continuous data on how zooplankton communities respond under the background of ecological restoration, and what characteristics they exhibit across different temporal and spatial scales, remain scarce.

In the present study, we integrated co-occurrence network topology analysis with spatiotemporal dynamics to reveal how their interactions reorganized under restoration, offering a mechanistic complement to traditional community indices. In addition, we analyzed the water quality and zooplankton community structure in inflow tributaries. These findings may inform targeted management (pollution interception) beyond generic reservoir recommendations. Surveys were conducted from 2021 to 2024, covering different regions, including the main inflows, central lacustrine zones and downstream region. By comparing two annual cycles during ecological restoration (the 2021 cycle and the 2023 cycle), we systematically analyzed the temporal and spatial dynamic characteristics of the zooplankton community in Shilianghe Reservoir and explored the potential driving mechanisms in combination with environmental factors. This study focuses on addressing the following questions: (1) Under the background of ecological restoration, what changes have occurred in the species composition and standing stock of the zooplankton community? (2) How have the diversity and topological characteristics of the co-occurrence network of the community changed? (3) What are the key aquatic environmental factors driving the changes in zooplankton community structure and distribution pattern? The findings aim to deepen the understanding of ecological processes in large shallow reservoirs, while providing a scientific basis for ecological management and evaluation methods for other restoration reservoirs.

## 2. Materials and Methods

### 2.1. Study Area and Sampling

**Figure 1:**
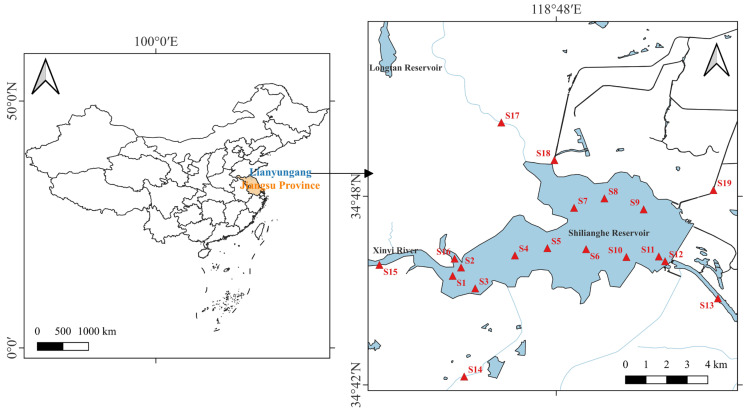
Location of sampling sites in the Shilianghe Reservoir.

Shilianghe Reservoir (34.7425–35.8294° N, 118.7364–118.8753° E) is located in the middle reaches of the Xinshu River and has a surface area of 60.8 km^2^ and an average water depth of 24.81 m. Accounting for environmental heterogeneity, this study selected five sampling regions (Figure 1): the tail region of the reservoir (upstream reservoir, S1–S4), where the hydrological regimes are complex and highly influenced by upstream rivers; the middle region of reservoir (mid-reservoir, S5–S8), where the hydrological regimes are relatively simple; the near-dam region of reservoir (near-dam reservoir, S9–S12); the dam downstream region (downstream of the dam, S13), 2 km from the outlet of the dam; and the inflowing river region (inflow tributaries, S14–S19), including the Longliang River (S14), Xinshu River (S15), Xizhufan River (S16), Shimentou River (S17), Fan River (S18) and Beigan Canal (S19) (Table A1).

Seasonal surveys of environmental factors and zooplankton communities were conducted in Shilianghe Reservoir in two sampling cycles (2021–2022 and 2023–2024), with sampling in July 2021 (summer), October 2021 (autumn), February 2022 (winter), May 2022 (spring), April 2023 (spring), July 2023 (summer), October 2023 (autumn), and January 2024 (winter). To compare the zooplankton community characteristics in different years, the data were grouped into two annual cycles: the 2021 sampling cycle (2021–2022) and the 2023 sampling cycle (2023–2024).

In this study, we collected 146 zooplankton samples and 146 water samples from 19 sites over two years (8 surveys), which can indicate sufficient sensitivity in reflecting spatiotemporal variations of the zooplankton community.

### 2.2. Environmental Factors Determination

The water transparency (SD) was measured at each sampling site using a Secchi disk (B0204017, Harbin Optical Instrument Factory, Harbin, China). The water temperature (WT), conductivity (Cond), dissolved oxygen (DO) and pH were measured with a multi-parameter water quality meter (Yellow Springs Instruments, Yellow Springs, OH, USA). The concentrations of total nitrogen (TN), total phosphorus (TP), potassium permanganate index (COD_Mn_) and chlorophyll a (Chl-a) were determined using a high-pressure steam sterilizer (GF54DA, ZEALWAY Instruments Co., Ltd., Wilmington, DE, USA), an ultraviolet spectrophotometer (UV-1800PC, Shanghai Mapada Instruments Co., Ltd., Shanghai, China) and a constant-temperature water bath (HH-8, YETUO Technology Co., Ltd., Shanghai, China) in accordance with standard methods [22]. In addition, the comprehensive trophic level index (TLI) of the water was calculated based on the monitoring indicators.

### 2.3. Zooplankton Collection and Analyses

For qualitative samples, a plankton net with a mesh size of 0.064 mm was used to collect Rotifera and Protozoa, while a net with a larger mesh size of 0.112 mm was employed for Copepoda and Cladocera. These qualitative samples were collected by slowly dragging nets in a “∞” pattern at a speed of 20–30 cm/s between the water surface and 0.5 m depth for 1–3 min. For quantitative samples, 20 L of mixed water samples from the upper, middle, and lower layers was collected using a plexiglass water sampler. Mixed water samples were filtered through plankton nets (sieve aperture 0.064 mm). Subsequently, qualitative and quantitative samples were transferred into 100 mL stoppered polyethylene bottles, which were immediately fixed with formaldehyde solution (Sinopharm Chemical Reagent Co., Ltd., Shanghai, China) and Lugol’s solution (Sinopharm Chemical Reagent Co., Ltd., Shanghai, China), respectively.

In the laboratory, microscopes (Sunny Optical Technology (Group) Co., Ltd., Yuyao, China) were used to identify the species, count the number of individuals, and measure the size of all zooplankton, from which biomass was calculated [23,24,25,26]. Zooplankton density was calculated as the number of zooplankton individuals per unit volume of water and expressed as individuals per liter (ind./L). To improve the reliability of counting results, three independent counts were performed for each sample. If there were significant differences in the counting results (i.e., the standard deviation of the three counts exceeded 15% of the average count), the sample was recounted.

### 2.4. Data Processing and Analysis

All data analysis and visualization were performed in R 4.4.1, using packages such as “vegan”, “ggplot2”, “ggpubr” and “igraph” [27,28,29,30]. The differences in zooplankton density, biomass, and α-diversity indices among years, seasons, and spatial regions were compared separately. Data normality was assessed using the Shapiro–Wilk test, and homogeneity of variance was evaluated using Levene’s test. For two-group comparisons, Student’s *t*-test was used when the data satisfied the assumptions of normal distribution and homogeneity of variance; otherwise, the Wilcoxon rank-sum test was applied. For comparisons among more than two groups, one-way analysis of variance (ANOVA) was used when these assumptions were met; otherwise, the Kruskal–Wallis test was applied. For variables showing significant differences among multiple groups, post hoc pairwise comparisons were performed using Tukey’s HSD test or Dunn’s test, and the *p* values were adjusted using the Benjamini–Hochberg false discovery rate (FDR) method to control for multiple testing. Statistical significance was set at *p* < 0.05.

To describe the composition, biodiversity, and structure of the zooplankton community, we calculated the indices of *α*-diversity with the Shannon–Weiner index (*H′*) [31], Pielou index (*J′*) [32], Margalef index (*D*) [33] and Simpson index (*S′*) [34] using the following formulas:

(1) $$H\prime \;=\;-\sum_{i=1}^{s}\left(\frac{n_{i}}{N}\right)\ln\left(\frac{n_{i}}{N}\right)$$

(2) $$J\prime =\frac{H\prime }{\ln S}$$

(3) $$D=\frac{\left(S-1\right)}{\ln N}$$

(4) $$S\prime =1\;-\;\sum_{i=1}^{s}{\left(\frac{n_{i}}{N}\right)}^{2}$$

where *S* is the number of species, *n* is the number of individuals of each species, and *N* is the total number of individuals.

To reduce the influence of extremely abundant species on community ordination, the zooplankton species density matrix was Hellinger-transformed prior to analysis. Bray–Curtis dissimilarity was calculated based on the transformed species matrix, and principal coordinate analysis (PCoA) was performed to visualize differences in zooplankton community composition among years, seasons and spatial regions. Permutational multivariate analysis of variance (PERMANOVA) with 999 permutations was used to test the effects of year, season and spatial region on zooplankton community structure. To assess whether the PERMANOVA results were influenced by differences in within-group multivariate dispersion, homogeneity of multivariate dispersion among years, seasons, and spatial regions was further tested using PERMDISP with 999 permutations. Pairwise PERMANOVA and pairwise PERMDISP comparisons were further conducted to evaluate whether significant group differences were mainly associated with shifts in community composition or differences in within-group dispersion. Year effects were also tested within each season to further assess the robustness of inter-annual differences.

Co-occurrence networks were constructed based on standardized zooplankton density data. Spearman correlation analysis was used to evaluate interspecific associations, and *p* values were adjusted for multiple testing using the false discovery rate (FDR) method. Only species pairs with strong and statistically significant correlations were retained as network edges, using the criteria |ρ| ≥ 0.5 and FDR-adjusted *p* < 0.05. This threshold was selected to retain associations with at least moderate correlation strength while reducing false-positive links caused by weak correlations and multiple comparisons [13]. Network topological properties (network density, modularity, average clustering coefficient, average path length and global efficiency) were quantified in the “igraph” R package.

The trophic status of the water quality was evaluated using the comprehensive trophic level index (*TLI*). *TLI* was calculated and classified using the following formula [35]:

(5) $$\mathrm{TLI}(\sum )=\;\sum W_{j}\;\times \;\mathrm{TLI}(j)$$

where *W_j_* represents the correlation weight of the trophic level index of the j-th parameter, and *TLI*(*j*) represents the trophic level index of the j-th parameter. With Chl-a as the reference parameter, the correlation weight of the j-th parameter was calculated by the following formula:

(6) $$W_{j}\;=\;\frac{r_{\mathrm{ij}}^{2}}{\sum_{j=1}^{m}r_{\mathrm{ij}}^{2}}$$

where *r_ij_* is the correlation coefficient between the j-th parameter and the reference parameter (Chl-a), and *m* is the number of evaluation parameters.

A series of continuous numbers from 0 to 100 were used to classify the trophic status of the water quality: *TLI* < 30 for oligotrophic, 30 < *TLI* ≤ 50 for mesotrophic, 50 < *TLI* ≤ 60 for slightly eutrophic, 60 < *TLI* ≤ 70 for moderately eutrophic, and *TLI* > 70 for highly eutrophic.

To reduce the influence of rare and occasional taxa on the stability and interpret-ability of constrained ordination, low-abundance and low-frequency species were excluded prior to db-RDA analysis. The filtered species density matrix was log(x + 1)-transformed, and distance-based redundancy analysis (db-RDA) was then performed based on Bray–Curtis dissimilarity to evaluate the relationships between zooplankton community composition and environmental variables. All environmental variables were standardized using Z-score transformation before analysis, and multi-collinearity was assessed using the variance inflation factor (VIF). Only variables with VIF < 10 were retained in the final model. The overall significance of the db-RDA model, constrained axes and marginal effects of individual environmental variables were evaluated using 999 permutation tests. The adjusted R^2^ of the model and the independent explanatory contribution of each environmental variable were calculated using the R packages “vegan” and “rdacca.hp”, respectively.

## 3. Results

### 3.1. Species Composition, Density and Biomass of Zooplankton

#### 3.1.1. Species Composition

**Figure 2:**
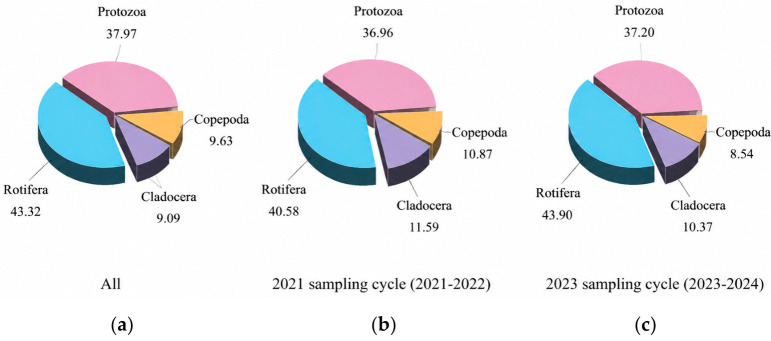
Zooplankton species composition: (**a**) zooplankton species composition from 2021 to 2024; (**b**) zooplankton species composition in the 2021 sampling cycle (2021–2022); (**c**) zooplankton species composition in the 2023 sampling cycle (2023–2024). Values indicate the percentage contribution of each zooplankton taxonomic group to the total number of recorded species (%).

A total of 187 zooplankton species from four taxa were identified from 2021 to 2024, including Protozoa (71 species, 37.97%), Rotifera (81 species, 43.32%), Cladocera (17 species, 9.09%) and Copepoda (18 species, 9.63%) (Figure 2).

**Figure 3:**
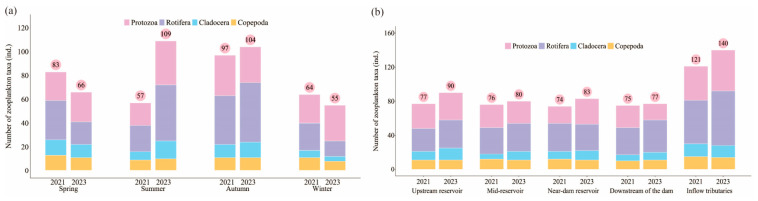
Annual and spatial variation in the taxonomic composition of zooplankton communities: (**a**) the number of zooplankton species in different years; (**b**) the number of zooplankton species in different regions.

Temporally, a total of 138 zooplankton species from four taxa were recorded in the 2021 sampling cycle, including Protozoa (51 species), Rotifera (56 species), Cladocera (16 species) and Copepoda (15 species) (Figure 2b). In the 2023 sampling cycle, a total of 164 zooplankton species from four taxa were collected, including Protozoa (61 species), Rotifera (72 species), Cladocera (17 species) and Copepoda (14 species) (Figure 2c). As the ecological restoration process progressed, the total number of zooplankton species increased, with the species of Protozoa and Rotifera increasing markedly, while the species number of Cladocera and Copepoda remained relatively stable (Figure 3a).

Spatially, 106, 101, 107 and 101 zooplankton species were recorded in the upstream reservoir, middle reservoir, near-dam reservoir and downstream of the dam, respectively. The zooplankton species reached 168 in the inflow tributaries, which was significantly higher than other regions (*p* < 0.05) (Figure 3b).

#### 3.1.2. Density and Biomass

**Figure 4:**
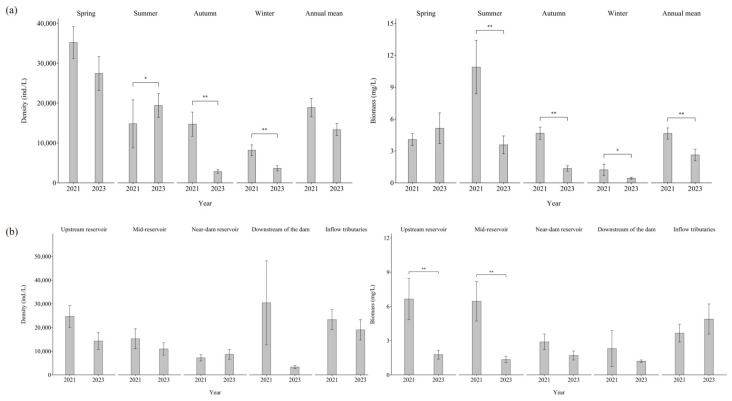
Annual and spatial variation in density and biomass of zooplankton: (**a**) comparison of zooplankton density and biomass in different years; (**b**) comparison of zooplankton density and biomass in different regions. Asterisks indicate significance levels: * *p* < 0.05; ** *p* < 0.01.

Overall, the density and biomass of zooplankton in the 2023 sampling cycle were lower than those in the 2021 sampling cycle, with a more pronounced decline in biomass (Table A2, Figure 4). The annual average density decreased from 18,904.35 ± 2296.87 ind./L in the 2021 sampling cycle to 13,341.74 ± 1523.44 ind./L in the 2023 sampling cycle, but the difference was only marginally significant (*p* = 0.052). In contrast, the annual average biomass decreased significantly from 4.65 ± 0.53 mg/L to 2.63 ± 0.54 mg/L (*p* = 0.001).

Both the density and biomass of zooplankton exhibited pronounced inter-annual differences in the same season (Figure 4a). In the summer of the 2023 sampling cycle, the density was significantly higher than that in the 2021 sampling cycle (*p* < 0.05), while in autumn and winter, it was significantly lower than that in the 2021 sampling cycle (*p* < 0.001). No significant difference was observed in spring between the two years (*p* > 0.05). Biomass was significantly lower in summer, autumn, and winter of the 2023 sampling cycle compared to the 2021 sampling cycle (*p* < 0.05), with the most pronounced decline occurring in summer. The difference in biomass in spring was not significant between the two years (*p* > 0.05).

No significant inter-annual differences in zooplankton density were observed across all regions (*p* > 0.05) (Figure 4b). Biomass decreased significantly in the upstream reservoir and middle reservoir (*p* < 0.05), while no significant changes were found in the near-dam reservoir, the downstream of the dam region, or the inflow tributaries (*p* > 0.05).

Notably, in addition to the decline in absolute biomass, mesozooplankton also exhibited a significant decreasing trend in relative biomass between years. Specifically, Cladocera and Copepoda accounted for a higher proportions of total biomass in summer, autumn, and winter of the 2021 sampling cycle, whereas their relative contributions decreased markedly in the 2023 sampling cycle (Table A2).

### 3.2. Diversity Analysis of Zooplankton

#### 3.2.1. The *α*-Diversity Index

The ranges of the four *α*-diversity indices (Margalef richness index (*D*), Shannon–Wiener diversity index (*H′*), Pielou evenness index (*J′*) and Simpson diversity index (*S′*)) were 0.62–3.78, 0.60–2.60, 0.25–0.88 and 0.25–0.90, respectively (Table A3 and Table A4).

Temporally, only *D* showed a significant difference between the 2021 sampling cycle and the 2023 sampling cycle in spring (*p* < 0.05) (Table A3). In summer, none of the diversity indices showed significant differences between the two years (*p* > 0.05). Autumn exhibited the most pronounced inter-annual differences in diversity. *H′*, *J′*, and *S′* were all significantly lower in the 2023 sampling cycle than in the 2021 sampling cycle (*p* < 0.05), while *D* showed no significant change. In winter, only *J′* showed a significant difference, with 2023 being significantly higher than 2021 (*p* < 0.05), while the other indices showed no significant differences (*p* > 0.05).

Spatially, only *D* and *H′* showed significant differences among different regions (*p* < 0.05) (Table A4). Generally, the *D* and *H′* in the upstream reservoir were higher than those in the near-dam reservoir.

#### 3.2.2. The *β*-Diversity Index

**Figure 5:**
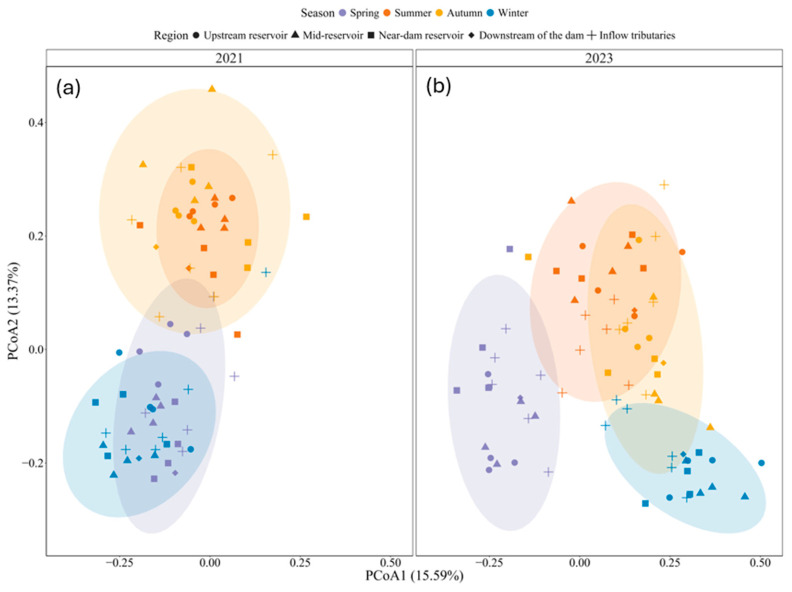
Principal coordinates analysis (PCoA) of zooplankton community composition in different years and regions. (**a**) Principal coordinates analysis (PCoA) of zooplankton community composition in the 2021 sampling cycle; (**b**) Principal coordinates analysis (PCoA) of zooplankton community composition in the 2023 sampling cycle.

Based on PCoA, the seasonal separation characteristics of zooplankton were more significant in the 2023 sampling cycle than in the 2021 sampling cycle (Figure 5). The results of the PERMANOVA analysis showed that the differences in zooplankton community structure among the four seasons were the most significant (R^2^ = 0.184, *p* = 0.001), followed by sampling year (R^2^ = 0.059, *p* = 0.001) and region (R^2^ = 0.035, *p* = 0.004) (Table A5).

PERMDISP analysis further showed no significant difference in multivariate dispersion among spatial regions (F = 0.351, *p* = 0.857), suggesting that the regional differences detected by PERMANOVA mainly reflected differences in community composition rather than dispersion heterogeneity (Table A5). In contrast, significant differences in multivariate dispersion were detected between years and among seasons (year: F = 9.043, *p* = 0.006; season: F = 10.691, *p* = 0.001), indicating that season- and year-related differences may partly reflect variation in within-group heterogeneity.

To further evaluate this issue, pairwise PERMANOVA was conducted for seasonal comparisons. All seasonal pairs showed significant differences in community composition (all *p* = 0.001). Notably, spring vs. summer and autumn vs. winter remained significant while showing no significant differences in multivariate dispersion, indicating that seasonal differences were not solely attributable to dispersion heterogeneity. In addition, year effects were examined within each season. PERMANOVA showed significant differences between 2021 and 2023 within all four seasons (all *p* = 0.001), whereas PERMDISP detected no significant year-related dispersion differences within any season. These results suggest that inter-annual differences in zooplankton community structure reflected consistent shifts in community composition rather than being driven only by differences in dispersion.

### 3.3. Co-Occurrence Networks of Zooplankton Community

**Figure 6:**
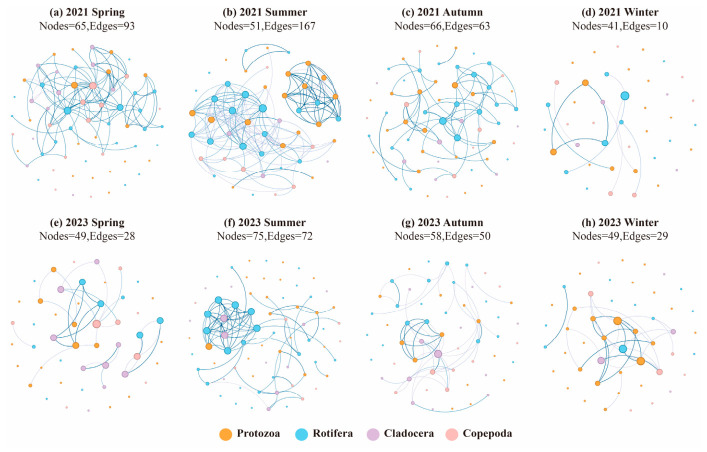
Co-occurrence networks of zooplankton communities in different sampling times.

**Figure 7:**
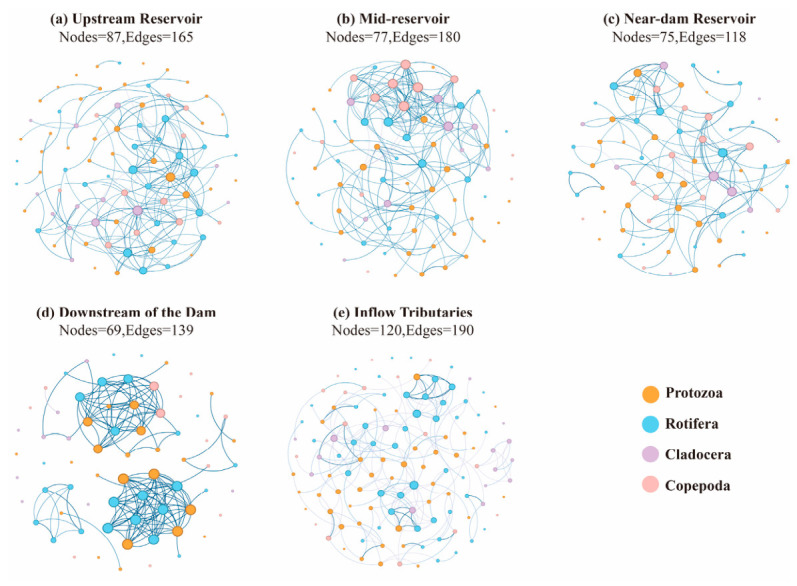
Co-occurrence networks of zooplankton communities in different regions.

**Figure 8:**
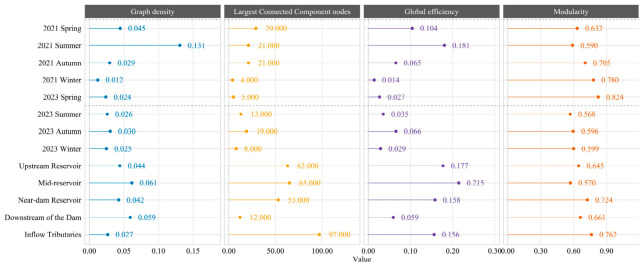
Comparison of network topological properties in different years and regions.

Based on the density data of species, zooplankton co-occurrence networks were constructed for each season and region. The networks showed obvious differences in zooplankton communities at both temporal and spatial scales (Figure 6 and Figure 7). Modularity remained at a high level (0.57–0.82) across all temporal and spatial networks, indicating that species associations exhibit a clear modular structure (Figure 8).

Temporally, compared with the 2021 sampling cycle, the network density in spring and summer of the 2023 sampling cycle decreased significantly, while the size of the largest connected component also shrank markedly, indicating obvious changes in network structure. In contrast, the network changes in autumn and winter were relatively small (Figure 6).

Spatially, the network density of zooplankton communities was highest in the mid-reservoir area (0.062), indicating closer associations among species. Although the inflow tributaries had a lower network density (0.027), they possessed the largest connected component, suggesting that more species participated in the core network, but the overall connections were relatively loose (Figure 7).

### 3.4. Relationship Between Zooplankton Community Structure and Environmental Factors

#### 3.4.1. Environmental Factors

**Figure 9:**
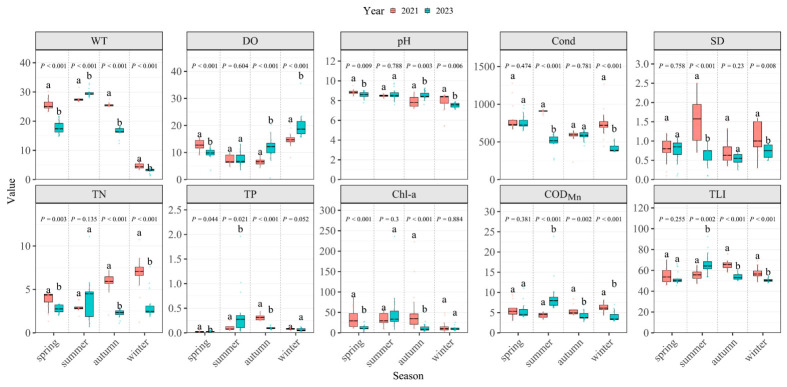
Temporal variations in the water quality indicators. A and b represent that there are significant differences in the water quality indicators of the four seasons. Abbreviations: WT, water temperature; DO, dissolved oxygen; pH, potential of hydrogen; Cond, conductivity; SD, water transparency; TN, total nitrogen; TP, total phosphorus; Chl-a, chlorophyll a; COD_Mn_, potassium permanganate index; TLI, trophic level index. Groups sharing at least one common letter are not significantly different, whereas groups without common letters differ significantly at *p* < 0.05.

Temporally, compared with the 2021 sampling cycle, most water quality indicators in the same seasons showed significant differences in the 2023 sampling cycle (*p* < 0.05). Overall, total nitrogen (TN), total phosphorus (TP), chlorophyll a (Chl-a), the potassium permanganate index (COD_Mn_), and the trophic level index (TLI) showed decreasing trends in multiple seasons (Figure 9).

**Figure 10:**
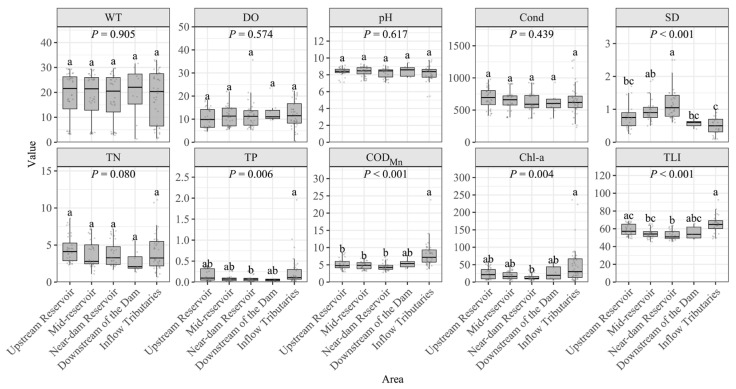
Regional variations in the water quality indicators. a, b and c represent that there are significant differences in the water quality indicators of the five regions. Abbreviations: WT, water temperature; DO, dissolved oxygen; pH, potential of hydrogen; Cond, conductivity; SD, water transparency; TN, total nitrogen; TP, total phosphorus; Chl-a, chlorophyll a; COD_Mn_, potassium permanganate index; TLI, trophic level index. Groups sharing at least one common letter are not significantly different, whereas groups without common letters differ significantly at *p* < 0.05.

Spatially, Secchi depth (SD), total phosphorus (TP), chlorophyll a (Chl-a), the potassium permanganate index (COD_Mn_) and the comprehensive trophic level index (TLI) showed significant differences among different regions (*p* < 0.05) (Figure 10). The water quality conditions were relatively better in the middle reservoir region and near-dam reservoir region, exhibiting a lightly eutrophic level.

#### 3.4.2. Relationship Between Zooplankton Community Structure and Environmental Factors

**Figure 11:**
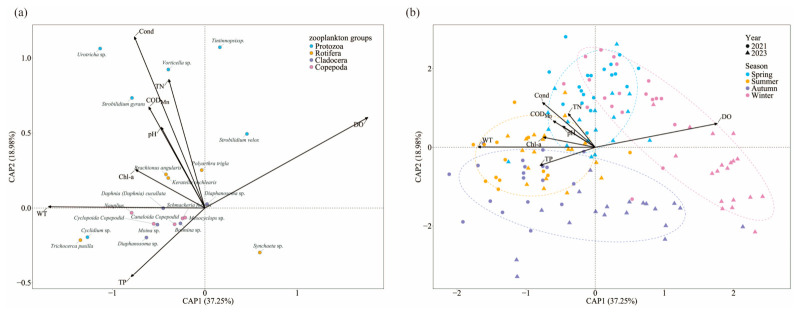
db-RDA analysis of the relationships between zooplankton and environmental factors. (**a**) Ordination relationships between zooplankton species and environmental factors; (**b**) ordination relationships between samples from different years and seasons and environmental factors. Percentages shown on the axes represent the proportion of constrained variation explained by each db-RDA axis. Abbreviations: WT, water temperature; DO, dissolved oxygen; pH, potential of hydrogen; Cond, conductivity; SD, water transparency; TN, total nitrogen; TP, total phosphorus; Chl-a, chlorophyll a; COD_Mn_, potassium permanganate index; TLI, trophic level index.

The relationships between zooplankton abundance and environmental parameters under the influence of ecological restoration measures are shown in Figure 11. The db-RDA analysis indicated that environmental factors significantly explained the variation in zooplankton community structure (*p* = 0.001). The measured environmental variables accounted for 11.88% of the total community variation. Among the constrained variation, the first two ordination axes (CAP1 and CAP2) explained 37.25% and 18.98%, respectively, accounting for 56.22% of the constrained variation in total. The marginal effects test indicated that water temperature (WT), dissolved oxygen (DO), total nitrogen (TN), and the potassium permanganate index (COD_Mn_) had the most significant independent explanatory effects on community structure (*p* = 0.001). Hierarchical partitioning analysis (rdacca.hp) showed that the main environmental factors affecting changes in the zooplankton community were dissolved oxygen (DO), water temperature (WT), conductivity (Cond) and total nitrogen (TN), with contribution rates of 27.72%, 23.27%, 10.99% and 10.54%, respectively.

Species ordination results showed that different dominant zooplankton species exhibited differentiated distributions along the environmental gradient (Figure 11a). Protozoa were more consistent with the direction of environmental gradients such as conductivity (Cond), total nitrogen (TN), and potassium permanganate index (COD_Mn_). Rotifera were correlated with water temperature (WT) and chlorophyll a (Chl-a), while Cladocera and Copepoda were mainly associated with water temperature (WT) and total phosphorus (TP), indicating that different zooplankton groups respond significantly differently to environmental factors.

The sample ordination results showed that the zooplankton community structures from different years exhibited some overlap in the ordination space, indicating that inter-annual differences existed. However, seasonal differentiation might be the main manifestation of community variation in Shilianghe Reservoir (Figure 11b).

## 4. Discussion

### 4.1. Inter-Annual Reorganization of the Zooplankton Community Under the Background of Ecological Restoration

Zooplankton are sensitive indicators of environmental change and food-web regulation in freshwater ecosystems [36,37]. This study found that the zooplankton community in Shilianghe Reservoir exhibited clear inter-annual reorganization, rather than a simple increase or decrease in standing stock during the advancement of ecological restoration. Compared with the 2021 sampling cycle, the zooplankton density and biomass in the 2023 sampling cycle decreased significantly, while the total number of species increased (especially in summer and autumn). This pattern suggests zooplankton community reorganization from high standing stock toward a richer but less biomass-dominated assemblage. This inter-annual reorganization is temporally consistent with the initial advancement of ecological restoration in Shilianghe Reservoir, possibly due to measures such as removal of aquaculture cages, control of external pollution, and regulation of fishery resources, which have reduced nutrient and organic matter inputs. Shilianghe Reservoir has a long history of intensive cage aquaculture (reaching approximately 100,000 cages by 2020). Cage aquaculture continuously imported large quantities of residual feed, feces and other organic matters into the reservoir. Aquaculture pollution from cage farming was an important source driving nutrient accumulation and increased organic loading in Shilianghe Reservoir [20,38,39]. With the removal of over 90% of cages by June 2021, along with measures such as pollution interception, the input of endogenous pollution into the reservoir has significantly decreased. Compared to 2021–2022, nutrient concentrations in the Shilianghe Reservoir were lower in 2023–2024, which corroborates the role of ecological restoration in improving water quality. The reduction in nutrients in the water is unfavorable for phytoplankton growth, which may in turn lead to a decline in zooplankton resources [40]. The improvement in aquatic environment may weaken the competitive advantage of pollution-tolerant zooplankton and provide niches for sustaining more species, particularly Protozoa and Rotifera, which are characterized by small body size, short generation and rapid reproduction rates [41,42,43,44].

In addition to an overall declining trend in biomass, the proportions of biomass contributed by different zooplankton taxa in the Shilianghe Reservoir also changed significantly. In the 2021 sampling cycle, Cladocera and Copepoda accounted for relatively high proportions of the total biomass in summer, autumn, and winter, whereas their relative contribution decreased substantially in the 2023 sampling cycle. This reveals that the prominent reduction in total biomass was mainly associated with the decline in mesozooplankton. Zooplankton are the primary feed source for larval stages of fish and filter-feeding fish such as silver carp and bighead carp [45,46,47,48]. A high density of these fish may increase the feeding pressure on zooplankton, especially mesozooplankton, thus reducing the biomass of the community and driving the zooplankton community toward miniaturization [49,50,51]. Since September 2021, the reservoir management department has released 35 million filter-feeding fish (silver carp and bighead carp) into the reservoir. The decline in both biomass and biomass proportion of mesozooplankton in this study is consistent with the potential food-web regulation effects induced by the stocking of silver carp and bighead carp. Thus, this phenomenon might be connected with fish predation pressure. However, since our study did not investigate fish community structure or actual feeding intensity, this speculation can only be considered a reasonable ecological hypothesis.

The temporal dynamics of zooplankton in Shilianghe Reservoir are reflected not only in density and biomass but also in changes in diversity patterns. The overall *α*-diversity of zooplankton in the 2023 sampling cycle was higher than that in the 2021 sampling cycle. Given the improving trend in the water quality of Shilianghe Reservoir, this increase in zooplankton diversity may reflect a partial weakening of environmental filtering pressure during the ecological restoration period. The *β*-diversity analysis revealed that differences in zooplankton community diversity were the most significant among seasons (*p* = 0.001), followed by year (*p* = 0.001) and sampling regions (*p* = 0.004). Zooplankton are a group of organisms with rapid generational turnover and high sensitivity to seasonal environmental variations, resulting in substantial seasonal fluctuations in their community [52,53,54]. This study only conducted a two-year investigation on zooplankton during the early stage of ecological restoration in the Shilianghe Reservoir. The inter-annual variations were smaller than seasonal differences, yet distinct changes were still detected. We infer that this inter-annual variation may have been influenced by ecological restoration. However, factors such as climatic variability, nutrient pulses, and hydrological regimes may also exert important influences on zooplankton communities [55,56,57]. What is more, stochastic ecological processes (stochastic colonization, population drift, priority effects, etc.) also play a non-negligible role in the inter-annual dynamics of zooplankton, especially in systems with moderate disturbance intensity [58,59,60,61]. Therefore, the inter-annual reorganization of the zooplankton community in Shilianghe Reservoir is temporally consistent with the restoration process, which may represent a systemic response under the context of multiple environmental influences and ecological restoration.

Compared with the 2021 sampling cycle, the co-occurrence network density and the size of the largest connected component in the 2023 sampling cycle both decreased significantly in spring and summer, indicating that species co-occurrence associations became looser and the core network structure contracted. This change may be related to factors such as nutrient reduction and stocking of filter-feeding fish. The former reduces the intensity of resource competition [57], while the latter alters the species dominance pattern through selective predation on larger species [62], both of which may weaken deterministic interspecific interactions. However, the co-occurrence network in this study was constructed based on abundance correlations. The observed topological variations may also be caused by unmeasured environmental factors or natural fluctuations in species occurrence frequencies between two sampling years. Accordingly, the decrease in network density cannot fully reflect a reduction in the intensity of interspecific interactions. It is noteworthy that the modularity index of all networks remained at a high level (0.57–0.82), indicating a tendency toward niche differentiation within the community to reduce direct competition, thereby helping to maintain the stability of community structure [63].

The environmental ordination results revealed that the inter-annual reorganization of zooplankton was associated with multiple environmental gradients. According to the db-RDA analysis, the measured environmental factors can explain a substantial fraction of the zooplankton community variation. Dissolved oxygen (DO), water temperature (WT), conductivity (Cond), and total nitrogen (TN) represent the oxygen environment, thermal conditions, ion or water source characteristics, as well as nutrient supply and organic matter load, respectively [64,65,66,67,68]. These four indices were the main environmental factors affecting the zooplankton community structure in Shilianghe Reservoir. Therefore, the reorganization of the zooplankton community during the ecological restoration period may be the result of the combined effects of water quality improvement, seasonal environmental fluctuations, and potential food-web regulation.

### 4.2. Effects of Spatial Heterogeneity on Zooplankton Community Structure

Although seasonal processes outweighed spatial zonation in explaining community variation, the zooplankton community in Shilianghe Reservoir still exhibited distinct spatial heterogeneity. Notably, the species number, density and biomass of zooplankton in the inflow tributaries were generally higher than in other regions. Tributaries typically exhibit high habitat heterogeneity, more complex hydrodynamic conditions, and a continuous input of zooplankton from upstream water bodies [69,70,71]. These factors result in the convergence of taxa adapted to both lotic and lentic environments in tributaries, making it an important channel for external nutrients and species inputs, as well as the most prominent area for zooplankton aggregation in the reservoir-river transition region [71,72,73].

According to the analysis of water environmental parameters, the concentrations of COD_Mn_ and TLI remained at relatively high levels in the inflow tributaries, indicating that these rivers were important nutrient input regions. This pattern may be related to the long-term influences of surrounding industrial activities and agricultural non-point-source pollution [74,75]. Therefore, tributaries can enhance community diversity through the input of upstream zooplankton. Meanwhile, continuous external nutrient inputs may maintain high levels of nutrients and organic matter, thereby limiting water quality improvement in the reservoir and limiting the ecological restoration process.

Based on the analysis of co-occurrence networks, the inflow tributaries had a relatively large number of network nodes but a low connectivity density, indicating that the zooplankton community in this region was characterized by high species diversity but loose interspecific relationships. This phenomenon may be related to the continuous input of exogenous water and strong hydrodynamic disturbances [11,71]. The habitat conditions in the middle and near-dam reservoir were more stable than other regions, where the zooplankton species richness and standing stock were relatively lower, and the community structure was relatively simpler and more stable.

The spatial differences in water environmental factors explain why the responses to ecological restoration may vary among different regions of the Shilianghe Reservoir. The middle reservoir and upstream reservoir have relatively better water quality and lower zooplankton standing stock, whereas the inflow tributaries maintain higher biological abundance under stronger external influences. This spatial difference suggests that the ecological restoration effectiveness of large shallow reservoirs should not be evaluated solely by the whole reservoir, but rather by distinguishing different functional zones, with particular attention to inflow tributaries, as different zones may respond to restoration measures through distinct ecological pathways.

### 4.3. Recommendations for Management and Future Research

According to the water quality analysis results, the ecological restoration of Shilianghe Reservoir has achieved initial success, while multiple challenges still remain. To continuously improve the aquatic ecological environment of Shilianghe Reservoir, we propose three suggestions based on its current ecological status. Firstly, the inflow tributaries should be regarded as the key region of external pollution interception and ecological restoration. It is essential to strengthen the control of agricultural non-point-source pollution during the flood season, surrounding domestic sewage discharge, and upstream industrial pollution, so as to reduce the impact of external nutrients on the water quality of the reservoir [76]. Secondly, in terms of filter-feeding fish regulation, the stocking intensity and timing of silver carp and bighead carp should be dynamically evaluated in combination with seasonal variations, algal abundance, and changes in zooplankton community structure. Excessive grazing pressure of filter-feeding fish on large-sized zooplankton through predation should be avoided, thereby weakening their potential control effect on algae [3]. Finally, it is recommended to establish a monitoring system covering phytoplankton, zooplankton, fish and water quality parameters, so as to identify the ecosystem dynamics in the process of ecological restoration and provide a scientific basis for the subsequent reservoir management strategies.

Our findings raise several questions that warrant further investigation. Firstly, although we observed dynamics of zooplankton communities following ecological restoration, the mechanisms linking filter-feeding fish stocking, water quality improvement, and zooplankton dynamics remain correlative. Future studies should combine higher-frequency plankton monitoring with water quality and other hydrological measurements, fish community surveys, and food-web analyses to quantify the relative importance of fish stocking and nutrient reduction in zooplankton community structure. Secondly, our research was not for definitive long-term trend detection and revealed that interspecific interactions of zooplankton are context-dependent. We recommend integrating high-frequency sampling with molecular approaches (e.g., eDNA) to resolve fine-scale temporal dynamics and trophic interactions, particularly in inflow tributaries where external nutrient inputs and hydrodynamic disturbances persist. Finally, Shilianghe Reservoir represents a typical case of phased restoration combining pollution control and biomanipulation. Conducting comparative studies on multiple shallow reservoirs under different restoration strategies will help distinguish the local responses of Shilianghe Reservoir from the general patterns in the ecological restoration of large shallow reservoirs. Such an integrated monitoring framework would enhance the application of zooplankton communities as biological indicators for evaluating ecological restoration.

## 5. Conclusions

Based on the two annual sampling cycles (2021–2022 and 2023–2024) in Shilianghe Reservoir, it was revealed that the zooplankton community exhibited a clear reorganization during the early phase of ecological restoration. The zooplankton community was characterized by significantly lower density and biomass but higher *α*-diversity in the 2023 sampling cycle compared to the 2021 cycle, accompanied by reduced co-occurrence network density and a contracted largest connected component in spring and summer, indicating a shift from a biomass-dominated assemblage toward a more diverse but less tightly interacting community. Seasonal variation was the strongest driver of community structure, followed by sampling year and spatial region. The main environmental factors affecting zooplankton community variations were dissolved oxygen (DO), water temperature (WT), conductivity (Cond) and total nitrogen (TN). Inflow tributaries supported the highest species richness and standing stock but relatively low network density, highlighting their role as species reservoirs and priority areas for external pollution interception. These findings demonstrate that early-phase restoration measures might have jointly contributed to the observed community reorganization. These findings provide a mechanistic framework for using zooplankton as bioindicators in reservoir restoration assessments and offer transferable insights for the ecological management of thousands of eutrophic reservoirs undergoing similar restoration measures in the Huai River Basin and beyond.

## Data Availability

All data are available within the Further inquiries can be directed to the corresponding author.

## Abbreviations

The following abbreviations are used in this manuscript:

SD	Water Transparency
WT	Water Temperature
Cond	Conductivity
DO	Dissolved Oxygen
TN	Total Nitrogen
TP	Total Phosphorus
COD_Mn_	Potassium Permanganate Index
Chl-a	Chlorophyll a
TLI	Trophic Level Index

## Appendix A

**Table A1:** Coordinates of sampling sites in the Shilianghe Reservoir.

Regions	Sites	Coordinates
Upstream reservoir	S1	34.7578° N, 118.7450° E
	S2	34.7622° N, 118.7494° E
	S3	34.7511° N, 118.7569° E
	S4	34.7686° N, 118.7781° E
Mid-reservoir	S5	34.7725° N, 118.7953° E
	S6	34.7719° N, 118.8158° E
	S7	34.7939° N, 118.8094° E
	S8	34.7989° N, 118.8256° E
Near-dam reservoir	S9	34.7931° N, 118.8464° E
	S10	34.7678° N, 118.8372° E
	S11	34.7681° N, 118.8544° E
	S12	34.7656° N, 118.8578° E
Downstream of the dam	S13	34.7458° N, 118.8858° E
Inflow tributaries	S14	34.7044° N, 118.7511° E
	S15	34.7636° N, 118.7061° E
	S16	34.7675° N, 118.7506° E
	S17	34.8392° N, 118.7708° E
	S18	34.8175° N, 118.7975° E
	S19	34.8006° N, 118.8881° E

**Table A2:** Seasonal comparison of zooplankton biomass in different years.

Season	Taxon	Biomass in 2021 (mg/L)	Biomass in 2023 (mg/L)	Adjusted *p*-Value	Relative Biomass 2021 (%)	Relative Biomass 2023 (%)
Spring	Protozoa	0.91 ± 0.15	0.50 ± 0.06	0.0163	22.37%	9.76%
	Rotifera	2.35 ± 0.41	1.76 ± 1.09	0.0118	57.45%	34.29%
	Cladocera	0.14 ± 0.06	0.76 ± 0.46	0.0734	3.46%	14.75%
	Copepoda	0.68 ± 0.34	2.11 ± 0.66	0.0101	16.72%	41.20%
Summer	Protozoa	0.11 ± 0.04	0.38 ± 0.08	0.0056	1.03%	10.73%
	Rotifera	1.24 ± 0.53	1.08 ± 0.41	0.0734	11.35%	30.28%
	Cladocera	5.52 ± 1.70	1.15 ± 0.29	0.1427	50.71%	32.33%
	Copepoda	4.02 ± 1.13	0.95 ± 0.28	0.0056	36.91%	26.66%
Autumn	Protozoa	0.23 ± 0.08	0.05 ± 0.01	0.0003	5.03%	3.45%
	Rotifera	0.52 ± 0.18	0.32 ± 0.10	0.7703	11.22%	23.57%
	Cladocera	1.55 ± 0.24	0.41 ± 0.15	0.0056	33.21%	30.22%
	Copepoda	2.36 ± 0.35	0.58 ± 0.17	0.0010	50.54%	42.76%
Winter	Protozoa	0.14 ± 0.02	0.12 ± 0.03	0.1256	11.63%	26.62%
	Rotifera	0.33 ± 0.12	0.22 ± 0.04	0.1912	26.86%	49.68%
	Cladocera	0.16 ± 0.04	0.01 ± 0.01	<0.0001	13.17%	1.00%
	Copepoda	0.59 ± 0.42	0.10 ± 0.05	0.0056	48.35%	22.69%

**Table A3:** Statistical results of zooplankton *α*-diversity index in different years.

Diversity Indices	Spring		Summer		Autumn		Winter	
	2021	2023	2021	2023	2021	2023	2021	2023
Margalef richness index (*D*)	1.38–3.25 (1.97 ± 0.11) ^a^	1.24–2.04 (1.58 ± 0.06) ^b^	0.62–3.61 (2.34 ± 0.24)	1.22–3.15 (2.18 ± 0.09)	1.30–3.37 (2.40 ± 0.13)	1.33–3.34 (2.07 ± 0.13)	0.77–3.78 (1.67 ± 0.15)	0.70–2.61 (1.44 ± 0.10)
Shannon–Wiener diversity index (*H′*)	0.86–2.60 (1.61 ± 0.10)	0.90–2.24 (1.64 ± 0.08)	1.29–2.47 (1.93 ± 0.11)	0.93–2.46 (1.87 ± 0.08)	1.25–2.46 (1.90 ± 0.10) ^a^	0.60–2.09 (1.39 ± 0.09) ^b^	0.68–2.46 (1.63 ± 0.09)	0.91–2.27 (1.77 ± 0.09)
Pielou evenness index (*J′*)	0.31–0.72 (0.52 ± 0.03)	0.35–0.75 (0.58 ± 0.03)	0.55–0.73 (0.64 ± 0.02)	0.38–0.71 (0.60 ± 0.02)	0.45–0.71 (0.60 ± 0.02) ^a^	0.25–0.71 (0.49 ± 0.03) ^b^	0.27–0.80 (0.62 ± 0.03) ^b^	0.47–0.88 (0.70 ± 0.02) ^a^
Simpson diversity index (*S′*)	0.36–0.89 (0.67 ± 0.04)	0.54–0.87 (0.73 ± 0.02)	0.64–0.87 (0.78 ± 0.02)	0.47–0.87 (0.77 ± 0.02)	0.59–0.88 (0.78 ± 0.02) ^a^	0.25–0.83 (0.63 ± 0.04) ^b^	0.41–0.90 (0.74 ± 0.02)	0.43–0.89 (0.77 ± 0.02)

Groups sharing at least one common letter are not significantly different, whereas groups without common letters differ significantly at *p* < 0.05.

**Table A4:** Statistical results of zooplankton *α*-diversity index in different regions.

Diversity Indices	Upstream Reservoir	Mid-Reservoir	Near-Dam Reservoir	Downstream of the Dam	Inflow Tributaries
Margalef richness index (*D*)	0.84–3.61 (2.16 ± 0.12) ^a^	1.27–3.37 (1.84 ± 0.08) ^ab^	0.62–2.57 (1.66 ± 0.07) ^b^	1.58–3.18 (2.04 ± 0.20) ^ab^	0.70–3.78 (2.04 ± 0.11) ^ab^
Shannon–Wiener diversity index (*H′*)	0.99–2.47 (1.90 ± 0.07) ^b^	0.67–2.37 (1.65 ± 0.07) ^ab^	0.60–2.27 (1.59 ± 0.06) ^a^	1.13–2.28 (1.68 ± 0.15) ^ab^	0.68–2.60 (1.71 ± 0.07) ^ab^
Pielou evenness index (*J′*)	0.32–0.78 (0.63 ± 0.02)	0.31–0.78 (0.58 ± 0.02)	0.25–0.88 (0.59 ± 0.02)	0.43–0.74 (0.57 ± 0.04)	0.27–0.80 (0.59 ± 0.02)
Simpson diversity index (*S′*)	0.41–0.88 (0.78 ± 0.02)	0.32–0.86 (0.72 ± 0.03)	0.25–0.89 (0.72 ± 0.02)	0.57–0.88 (0.73 ± 0.04)	0.41–0.90 (0.72 ± 0.02)

Groups sharing at least one common letter are not significantly different, whereas groups without common letters differ significantly at *p* < 0.05.

**Table A5:** PERMANOVA and PERMDISP results for zooplankton community composition among seasons, years, and spatial regions.

Analysis	Comparison/Factor	R^2^	Adjusted *p*	Significance
PERMANOVA	Season	0.184	0.001	***
PERMANOVA	Year	0.059	0.001	***
PERMANOVA	River	0.035	0.004	**
PERMDISP	Season	—	0.001	***
PERMDISP	Year	—	0.006	**
PERMDISP	Region	—	0.857	ns
Pairwise PERMANOVA	Spring vs. Summer	0.18	0.001	***
Pairwise PERMDISP	Spring vs. Summer	—	0.741	ns
Pairwise PERMANOVA	Spring vs. Autumn	0.147	0.001	***
Pairwise PERMDISP	Spring vs. Autumn	—	0.001	***
Pairwise PERMANOVA	Spring vs. Winter	0.091	0.001	***
Pairwise PERMDISP	Spring vs. Winter	—	<0.001	***
Pairwise PERMANOVA	Summer vs. Autumn	0.089	0.001	***
Pairwise PERMDISP	Summer vs. Autumn	—	0.049	*
Pairwise PERMANOVA	Summer vs. Winter	0.144	0.001	***
Pairwise PERMDISP	Summer vs. Winter	—	0.001	***
Pairwise PERMANOVA	Autumn vs. Winter	0.126	0.001	***
Pairwise PERMDISP	Autumn vs. Winter	—	0.6	ns
Within-season PERMANOVA	Spring: 2021 vs. 2023	0.222	0.001	***
Within-season PERMDISP	Spring: 2021 vs. 2023	—	0.122	ns
Within-season PERMANOVA	Summer: 2021 vs. 2023	0.223	0.001	***
Within-season PERMDISP	Summer: 2021 vs. 2023	—	0.08	ns
Within-season PERMANOVA	Autumn: 2021 vs. 2023	0.222	0.001	***
Within-season PERMDISP	Autumn: 2021 vs. 2023	—	0.465	ns
Within-season PERMANOVA	Winter: 2021 vs. 2023	0.283	0.001	***
Within-season PERMDISP	Winter: 2021 vs. 2023	—	0.177	ns

Abbreviations: R^2^ indicates the proportion of variation explained by each factor in PERMANOVA. Significance levels are indicated as follows: *** *p* ≤ 0.001; ** *p* < 0.01; * *p* < 0.05; ns, not significant.

**Table A6:** Checklist of zooplankton species.

Category	Species	2021	2023
Protozoa	*Acanthocystis* sp.	+	+
	*Amoeba* sp.	+	+
	*Arcella discoides*	+	+
	*Arcella hemisphaerica*		+
	*Arcella hemisphaerica undulata*	+	
	*Arcella vulgaris*	+	
	*Askenasia volvox*	+	+
	*Centropyxis aculeata*	+	+
	*Centropyxis aculeata grandis*	+	+
	*Centropyxis ecornis*	+	+
	*Centropyxis ecornis leidyi*	+	
	*Chilodonella* sp.		+
	*Ciliate*		+
	*Coleps* sp.	+	+
	*Colpoda* sp.	+	
	*Condylostoma* sp.		+
	*Cyclidium* sp.	+	+
	*Cyphoderia ampulla*	+	+
	*D.balbianii*	+	+
	*D.balbianii rostratum*	+	
	*Didinium balbianii nanum*	+	+
	*Didinium nasutum*	+	+
	*Difflugia acuminata*		+
	*Difflugia ampora*		+
	*Difflugia avellana*		+
	*Difflugia elegans*	+	+
	*Difflugia glans*	+	+
	*Difflugia globulosa*		+
	*Difflugia gramen*	+	+
	*Difflugia mammillaris*	+	+
	*Difflugia oblonga*	+	+
	*Difflugia* sp.		+
	*Difflugia tuberspinifera*	+	+
	*Difflugia urceolata*	+	+
	*Dileptus* sp.	+	+
	*Enchelydium fusidens*	+	+
	*Epistylis lacustris*	+	
	*Epistylis* sp.		+
	*Euplotes* sp.	+	+
	*Frontonia* sp.	+	+
	*Glaucoma* sp.		+
	*Halteria grandinella*	+	
	*Hastatella radians Erlanger*		+
	*Holophrya atra*	+	+
	*Lacrymaria minima*	+	+
	*Lacrymaria* sp.	+	+
	*Lagynophrya* sp.		+
	*Litonotus* sp.	+	+
	*Mesodinium pulex*	+	
	*Microthorax* sp.		+
	*Nassula* sp.	+	+
	*Opisthotricha* sp.		+
	*Oxytricha* sp.	+	
	*Paramecium* sp.		+
	*Prorodon* sp.	+	+
	*Sphaerophrya* sp.	+	+
	*Stentor* sp.		+
	*Strobilidium gyrans*	+	+
	*Strobilidium* sp.		+
	*Strobilidium velox*	+	+
	*Strombidium* sp.		+
	*Tintinnidium fluviatile*	+	+
	*Tintinnidium pusillum*	+	+
	*Tintinnopsis lacutris*	+	+
	*Tintinnopsis orientalis*		+
	*Tintinnopsis sinensis*	+	+
	*Tintinnopsis wangi*	+	+
	*Tintinnopsis* sp.	+	+
	*Trachelophyllum pusillum*	+	
	*Urotricha* sp.	+	+
	*Vorticella* sp.	+	+
Rotifera	*Anuraeopsis fissa*	+	+
	*Asplanchna* sp.	+	+
	*Bdelloidea*		+
	*Brachionus angularis*	+	+
	*Brachionus budapestiensis*	+	+
	*Brachionus califlorus*	+	+
	*Brachionus capsuliflorus*	+	+
	*Brachionus caudatus*	+	+
	*Brachionus diversicornis Daday*	+	+
	*Brachionus falcatus*		+
	*Brachionus forficula*	+	+
	*Brachionus leydigi*	+	+
	*Brachionus urceus*	+	+
	*Cephalodella catellina*	+	+
	*Cephalodella exigna*	+	+
	*Cephalodella gibba*	+	
	*Cephalodella* sp.	+	+
	*Chromogaster* sp.	+	
	*Collotheca* sp.	+	+
	*Colurella adriatic*	+	
	*Colurella obtusa*		+
	*Colurella* sp.		+
	*Colurella unicauda*	+	+
	*Conochilus dossuarius*	+	+
	*Conochilus unicornis*	+	+
	*Conochilus* sp.		+
	*Epiphanes* sp.	+	+
	*Euchlanis dilatata*	+	+
	*Euchlanis* sp.		+
	*Filinia brachiata*	+	+
	*Filinia longiseta*	+	+
	*Filinia maior*		+
	*Filinia minuta*	+	+
	*Filinia passa*		+
	*Hexarthra mira*	+	+
	*Keratella cochlearis*	+	+
	*Keratella quadrata*	+	+
	*Keratella valga*	+	+
	*Lecane bulla*	+	+
	*Lecane closterocerca*	+	+
	*Lecane cornuta*		+
	*Lecane elachis*		+
	*Lecane hamata*	+	
	*Lecane hastata*		+
	*Lecane ludwigii*		+
	*Lecane luna*	+	+
	*Lecane lunaris*	+	+
	*Lecane papuana*	+	+
	*Lecane pyriformis*		+
	*Lecane* sp.	+	+
	*Lecane stenroosi*	+	+
	*Lecane thienemanni*		+
	*Lecane unguitata*		+
	*Lecane ungulata*		+
	*Lepadella acuminata*		+
	*Lepadella obtusa*		+
	*Lepadella patella*	+	+
	*Lophocharis oxysternon*		+
	*Mytilina ventralis*		+
	*Notholca labis*	+	+
	*Notholca squamula*	+	
	*Notommata* sp.		+
	*Philodina* sp.	+	+
	*Plationus patulus*		+
	*Polyarthra trigla*	+	+
	*Pompholyx sulcata*	+	+
	*Proales* sp.		+
	*Rhinoglena frontalis*	+	
	*Rotaria neptunia*	+	+
	*Scaridium longicaudum*	+	+
	*Synchaeta oblonga*	+	
	*Synchaeta* sp.	+	+
	*Testudinella patina*	+	+
	*Tetramastix opoliensis*	+	
	*Trichocerca cylindrica*		+
	*Trichocerca pusilla*	+	+
	*Trichocerca rousseleti*	+	
	*Trichocerca similis*	+	+
	*Trichocerca* sp.	+	+
	*Trichocerca tenuior*		+
	*Trichotria tetractis*	+	+
Cladocera	*Alona* sp.	+	+
	*Bosmina* sp.	+	+
	*Bosminopsis deitersi*	+	+
	*Camptocercus* sp.	+	+
	*Ceriodaphnia cornuta*	+	+
	*Chydorus* sp.	+	+
	*Daphnia* (*Daphnia*) *cucullata*	+	+
	*Daphnia* (*Daphnia*) *hyalina*		+
	*Diaphanosoma* sp.	+	+
	*Ilyocryptus* sp.	+	+
	*Leptodora kindtii*	+	+
	*Leydigia* sp.	+	+
	*Moina* sp.	+	+
	*Pleuroxus* sp.	+	+
	*Scapholeberis mucronata*	+	+
	*Sida crystallina*	+	+
	*Simocephalus* sp.	+	+
Copepoda	*Canaloida Copepodid*	+	+
	*Cyclopoida Copepodid*	+	+
	*Cyclops* sp.		+
	*Cyclops strenuuss*	+	
	*Ectocyclops* sp.	+	
	*Eucyclops* sp.	+	+
	*Harpacticoida* sp.	+	+
	*Limnoithona sinensis*	+	
	*Mesocyclops* sp.	+	+
	*Microcyclops* sp.		+
	Nauplius	+	+
	*Neodiaptomus schmackeri*	+	+
	*Paracyclops* sp.	+	+
	*Phyllodiaptomus tunguidus*	+	
	*Schmackeria forbesi*	+	+
	*Sinocalanus dorrii*	+	+
	*Sinocalanus* sp.		+
	*Thermocyclops* sp.	+	+

+ indicates that the species was detected.
